# Immunogenicity of Seven New Recombinant Yellow Fever Viruses 17D Expressing Fragments of SIVmac239 Gag, Nef, and Vif in Indian Rhesus Macaques

**DOI:** 10.1371/journal.pone.0054434

**Published:** 2013-01-15

**Authors:** Mauricio A. Martins, Myrna C. Bonaldo, Richard A. Rudersdorf, Shari M. Piaskowski, Eva G. Rakasz, Kim L. Weisgrau, Jessica R. Furlott, Christopher M. Eernisse, Marlon G. Veloso de Santana, Bertha Hidalgo, Thomas C. Friedrich, Maria J. Chiuchiolo, Christopher L. Parks, Nancy A. Wilson, David B. Allison, Ricardo Galler, David I. Watkins

**Affiliations:** 1 Department of Pathology, University of Miami Miller School of Medicine, Miami, Florida, United States of America; 2 Laboratório de Biologia Molecular de Flavivírus, Instituto Oswaldo Cruz – FIOCRUZ, Rio de Janeiro, Brazil; 3 Wisconsin National Primate Research Center, University of Wisconsin-Madison, Madison, Wisconsin, United States of America; 4 Department of Pathobiological Sciences, University of Wisconsin-Madison, Madison, Wisconsin, United States of America; 5 International AIDS Vaccine Initiative, AIDS Vaccine Design and Development Laboratory, Brooklyn Army Terminal, Brooklyn, New York, United States of America; 6 Department of Medicine, University of Wisconsin-Madison, Madison, Wisconsin, United States of America; 7 Section on Statistical Genetics, Department of Biostatistics, University of Alabama at Birmingham, Birmingham, Alabama, United States of America; University of Cape Town, South Africa

## Abstract

An effective vaccine remains the best solution to stop the spread of human immunodeficiency virus (HIV). Cellular immune responses have been repeatedly associated with control of viral replication and thus may be an important element of the immune response that must be evoked by an efficacious vaccine. Recombinant viral vectors can induce potent T-cell responses. Although several viral vectors have been developed to deliver HIV genes, only a few have been advanced for clinical trials. The live-attenuated yellow fever vaccine virus 17D (YF17D) has many properties that make it an attractive vector for AIDS vaccine regimens. YF17D is well tolerated in humans and vaccination induces robust T-cell responses that persist for years. Additionally, methods to manipulate the YF17D genome have been established, enabling the generation of recombinant (r)YF17D vectors carrying genes from unrelated pathogens. Here, we report the generation of seven new rYF17D viruses expressing fragments of simian immunodeficiency virus (SIV)mac239 Gag, Nef, and Vif. Studies in Indian rhesus macaques demonstrated that these live-attenuated vectors replicated *in vivo,* but only elicited low levels of SIV-specific cellular responses. Boosting with recombinant Adenovirus type-5 (rAd5) vectors resulted in robust expansion of SIV-specific CD8^+^ T-cell responses, particularly those targeting Vif. Priming with rYF17D also increased the frequency of CD4^+^ cellular responses in rYF17D/rAd5-immunized macaques compared to animals that received rAd5 only. The effect of the rYF17D prime on the breadth of SIV-specific T-cell responses was limited and we also found evidence that some rYF17D vectors were more effective than others at priming SIV-specific T-cell responses. Together, our data suggest that YF17D – a clinically relevant vaccine vector – can be used to prime AIDS virus-specific T-cell responses in heterologous prime boost regimens. However, it will be important to optimize rYF17D-based vaccine regimens to ensure maximum delivery of all immunogens in a multivalent vaccine.

## Introduction

The HIV epidemic is one of the worst public health tragedies in human history. More than 25 million human lives have been lost due to Acquired Immune Deficiency Syndrome (AIDS)-related causes since the first description of the disease in 1981 [1], [2]. Sadly, two thirds of the 33 million people currently infected with HIV infection live in resource-poor regions of the globe where access to standard healthcare is limited [2]. Despite advances in access to anti-retroviral treatment (ART) in countries with the heaviest burden of the disease, less than half of the population in need of this therapy actually receives it [2]. A safe and effective HIV vaccine is, therefore, the best long-term solution to ending the pandemic [3].

Several lines of evidence implicate CD8^+^ T-cells in the control of HIV-1 replication [4]–[10]. It is also becoming increasingly clear that lentivirus-specific CD4^+^ T-cells contribute to an effective antiviral immune response [11]–[14]. As a result, a myriad of vaccination platforms have been developed to induce HIV-specific cellular responses. Viral vectors are of particular interest since this strategy exploits the proficiency with which viruses enter cells and hijack the cellular machinery to promote maximal expression of viral proteins [15]. Non-replicating adenoviruses and poxviruses, for instance, have been heavily exploited as potential HIV vector systems due to their good preclinical immunogenicity and safety profiles [15]–[18]. However, the high prevalence worldwide of pre-existing immunity to Ad5 and the safety concerns raised by the rAd5-vectored HIV vaccine tested in STEP study have dampened the enthusiasm toward this vector platform [19]–[22]. Furthermore, modified vaccinia virus Ankara (MVA)- and canarypox (ALVAC) expressing HIV immunogens have elicited low levels of cellular immunity in most human clinical trials conducted so far [23]–[25]. Therefore, novel viral vectors capable of safely inducing robust T-cell responses in humans are needed to advance HIV vaccine design.

Historically, live-attenuated replicating viral vaccines have provided the most effective protection against infection and disease [18]. Notably, these vaccines elicit robust and durable immune responses in immunized individuals, which is rarely achieved by inactivated or subunit vaccines [18]. The live-attenuated yellow fever vaccine virus YF17D, for instance, is one of the most successful human vaccines ever developed. More than 400 million people have been vaccinated since its development in the 1930s, with only rare adverse events [19], [26]–[28]. Perhaps because it can replicate in humans, YF17D triggers several innate immune pathways, which likely contributes to its high immunogenicity [29], [30]. Indeed, vaccination results in polyvalent adaptive immune responses consisting of effector CD8^+^ T-cells and a mixed T_H_1/T_H_2 CD4^+^ T-cell profile [28]. Importantly, southern Africa and Asia – two regions with high incidence of HIV cases – are not considered endemic regions for yellow fever and thus vaccine coverage in these areas is low [31], [32]. As a result, there is a low prevalence of pre-existing immunity to the yellow fever virus in these important target populations. Therefore, vector-specific immunity is unlikely to affect the immunogenicity of a potential rYF17D/HIV vaccine in those areas. These properties make YF17D a promising vector platform for delivering HIV genes.

Several methods for manipulating the YF17D genome have been developed [33]–[35], including the generation of rYF17D viruses bearing insertions between the genes encoding Envelope (E) and non-structural protein 1 (NS1) [36]. We have recently used this methodology to create a recombinant YF17D virus expressing a segment of SIVmac239 Gag. This virus replicated and elicited CD8^+^ T-cell responses in rhesus macaques [37]. Here, we employed this same methodology to generate seven new rYF17D vectors expressing fragments of the SIVmac239 Gag, Nef, and Vif proteins. We chose these antigens since vaccine-induced T-cells targeting these proteins have been associated with control of viral replication after a pathogenic SIV challenge [38]. The goal of this study was, therefore, to evaluate the immunogenicity of these seven rYF17D vectors alone or as a prime in a heterologous prime/boost regimen.

## Materials and Methods

### Research Animals and Ethics Statement

The animals in this study were Indian rhesus macaques (*Macaca mulatta*) and were part of the breeding colony of the Wisconsin National Primate Research Center (WNPRC). All animals were cared for in accordance with the guidelines of the Weatherall report [39] under a protocol approved by the University of Wisconsin Graduate School Animal Care and Use Committee (animal welfare assurance no. A3368-01; protocol no. G00578). Furthermore, the macaques in this study were managed according to the animal husbandry program of the WNPRC, which aims at providing consistent and excellent care to nonhuman primates at the center. This program is employed by the Colony Management Unit and is based on the laws, regulations, and guidelines promulgated by the United States Department of Agriculture (e.g., the Animal Welfare Act and its regulations, and the Animal Care Policy Manual), Institute for Laboratory Animal Research (e.g., Guide for the Care and Use of Laboratory Animals, 8^th^ edition), Public Health Service, National Research Council, Centers for Disease Control, and the Association for Assessment and Accreditation of Laboratory Animal Care (AAALAC) International. The nutritional plan utilized by the WNPRC is based on recommendations published by the National Research Council. Specifically, macaques were fed twice daily with 2050 Teklad Global 20% Protein Primate Diet and food intake was closely monitored by Animal Research Technicians. This diet was also supplemented with a variety of fruits, vegetables, and other edible objects as part of the environmental enrichment program established by the Behavioral Management Unit. Paired/grouped animals exhibiting stereotypes and/or incompatible behaviors were reported to the Behavioral Management staff and managed accordingly. All primary enclosures (i.e., stationary cages, mobile racks, and pens) and animal rooms were cleaned daily with water and sanitized at least once every two weeks. All macaques used in this study were males, except for three females: r98010, r03105, and r04149. Their average weight was 8.0 kg (range: 6.5–9.8 kg) and their average age was 5 years (range: 4–11 years). Vaccinations were performed under anesthesia (Ketamine administered at 5–12 mg/kg depending on the animal) and all efforts were made to minimize suffering. None of the animals were euthanized as part of this study. The macaques were typed for common major histocompatibility complex (MHC) class I alleles by sequence-specific PCR analysis [40], [41] and were chosen based on the expression of Mamu-A*01 and/or Mamu-A*02 (Table 1).

**Table 1:** Animal details and vaccination doses.

Animal ID	MHC-I	rYF17D/SIV vectors	Dose	rAd5 vectors	Dose
r04147	Mamu-A*02	Gag (44–84)	10^5^ PFU	N.A.	N.A.
r04170	Mamu-A*01	Gag (76–123)	10^5^ PFU	N.A.	N.A.
r04136	Mamu-A*01	Gag (142–186)	10^5^ PFU	N.A.	N.A.
r05079	Mamu-A*01	Gag (250–415)	10^5^ PFU	N.A.	N.A.
r05089	Mamu-A*02	Vif (1–110)	10^5^ PFU	N.A.	N.A.
r04109	Mamu-A*01	Vif (102–214)	10^5^ PFU	N.A.	N.A.
r05070	Mamu-A*02	Nef (45–210)	10^5^ PFU	N.A.	N.A.
r05028	Mamu-A*01, A*02	All constructs	10^4^ PFU each	(i) Gag, (ii) Nef, and (iii) a fusion ofVif, Vpr, Vpx, Tat, and Rev	10^11^ VP each
rh2138	Mamu-A*02	All constructs	10^4^ PFU each	(i) Gag, (ii) Nef, and (iii) a fusion ofVif, Vpr, Vpx, Tat, and Rev	10^11^ VP each
r04137	Mamu-A*01, A*02	All constructs	10^5^ PFU each	(i) Gag, (ii) Nef, and (iii) a fusion ofVif, Vpr, Vpx, Tat, and Rev	10^11^ VP each
r98010	Mamu-A*02	All constructs	10^5^ PFU each	(i) Gag, (ii) Nef, and (iii) a fusion ofVif, Vpr, Vpx, Tat, and Rev	10^11^ VP each
r03105	Mamu-A*01	N.A.	N.A.	(i) Gag, (ii) Nef, and (iii) a fusion ofVif, Vpr, Vpx, Tat, and Rev	10^11^ VP each
r04149	Mamu-A*01	N.A.	N.A.	(i) Gag, (ii) Nef, and (iii) a fusion ofVif, Vpr, Vpx, Tat, and Rev	10^11^ VP each
r05073	Mamu-A*02	N.A.	N.A.	(i) Gag, (ii) Nef, and (iii) a fusion ofVif, Vpr, Vpx, Tat, and Rev	10^11^ VP each
r05086	Mamu-A*01	N.A.	N.A.	(i) Gag, (ii) Nef, and (iii) a fusion ofVif, Vpr, Vpx, Tat, and Rev	10^11^ VP each

N.A., not applicable.

### Generation of Recombinant YF17D Viruses Expressing Fragments of SIVmac239 Proteins

We designed seven codon-optimized minigenes encoding fragments of the SIVmac239 Gag, Nef, and Vif proteins. Four of these encoded segments of the Gag precursor protein (amino acids 44–84, 76–123, 142–186, and 250–415); two encoded the amino and carboxyl portions of Vif (1–110 and 102–214); and one encoded the central part of Nef (45–210). We generated viable recombinant YF17D viruses expressing these protein fragments using a previously described methodology [36]. Briefly, we duplicated functional motifs flanking the E and NS1 intergenic regions in the YF17D backbone and fused them to the exogenous minigenes, allowing for the correct processing of the viral polyprotein precursor. The recombinant SIV protein fragments are released into the lumen of the endoplasmic reticulum via the action of host peptidases. After cloning the inserts into the junction of E and NS1, we prepared full-length rYF17D genomic RNA by *in vitro* transcription using SP6 RNA polymerase (AmpliScribe SP6, Epicentre Technologies). These RNA preparations were used to transfect Vero cells with LipofectAmine (Invitrogen) as described elsewhere [42]. Viral stocks were prepared by infecting Vero cell monolayers with the virus present in the supernatant from the transfections described above.

To confirm the presence and integrity of SIV inserts, we extracted viral RNA (vRNA) from culture supernatants (QIAmp Viral RNA kit, QIAGEN) and performed an amplification of the viral E-NS1 genomic region encompassing the heterologous insert. For the cDNA synthesis (SuperScript III Reverse Transcriptase, Invitrogen), we used viral RNA as a template and a negative strand YF17D-specific synthetic oligonucleotide (genome position 2772–2795). Subsequently, we performed PCR (Master Mix Green Go Taq, Promega) in the presence of a positive strand YF17D-specific primer (genome position 2122–2145). Amplification products were further purified from excess primers with silica-based kits (QIAGEN) and run on an agarose gel.

### YF17D Viral Load Assay

We measured the replication of rYF17D viruses by isolating vRNA from EDTA-anticoagulated plasma by proteinase K digestion of the nucleocapsid in the presence of guanidine hydrochloride as described previously [43]. Viral RNA was reverse transcribed and amplified using the SuperScript III Platinum® One-Step Quantitative RT-PCR System (Invitrogen, Carlsbad, CA) in a Roche LightCycler® 480. The final reaction mixtures (100-µl total volume) contained 0.2 mM (each) deoxynucleoside triphosphates, 3.5 mM MgS0_4_, 750 ng of random hexamer primers (Promega, Madison, WI), 4.0 µl of SuperScript III reverse transcriptase and Platinum Taq DNA polymerase in a single enzyme mix, 600 nM (each) amplification primers (forward [YF-17D 10188], 5′-GCGGATCACTGATTGGAATGAC-3′, and reverse [YF-17D 10264], 5′-CGTTCGGATACGATGGATGACTA-3′), and 100 nM probe [6Fam]5′-AATAGGGCCACCTGGGCCTCCC-3′[TamraQ]. The reverse transcription reaction was performed at 37°C for 15 minutes and then at 50°C for 30 minutes. Taq polymerase was activated subsequently by incubation at 95°C for 2 minutes after which 50 amplification cycles were performed at 95°C for 15 seconds and 57°C for one minute with ramp times set to 2.2°C/s. Ten-fold serial dilutions of a YF17D *in vitro* transcript were used to generate a standard curve for each run. The transcript was made using a MEGAscript® (Ambion, Austin, TX) high yield transcription kit. The plasmid, pCR-Blunt, containing a PCR amplicon with a sequence identical to nucleotides 8621–10354 of GenBank accession number U17066, served as the template for transcription. The limit of detection under standard assay conditions was approximately 45 vRNA copies/ml of plasma.

### Vaccination of Rhesus Macaques with rYF17D and rAd5 Vectors

We immunized animals with rYF17D viruses via the subcutaneous route. For recipients of single constructs, we injected 10^5^ plaque-forming units (PFU) diluted with PBS to 500 µl into the animals’ right forearm. For recipients of all constructs, we injected either 10^4^ or 10^5^ PFU of each rYF17D virus (using 27-gauge needles) into seven different sites; right and left thighs; right and left forearms; right and left shoulders; and left lower leg.

We subsequently boosted the four animals that received all seven rYF17D viruses (either at 10^4^ or 10^5^ PFU of each) with recombinant Adenovirus type-5 (rAd5). We used three rAd5 vectors encoding full-length (i) Gag, (ii) Nef, and (iii) a fusion of the Vif, Tat, Rev, Vpr and Vpx proteins of SIVmac239. The rAd5/Gag vector encoded a myristoylated Gag protein spanning amino acids 1 to 508, lacking two C-terminal amino acids. The rAd5/Nef vector encoded Nef lacking a myristoylation signal. The rAd5 vectors were constructed as described previously [44]. Briefly, they were generated in a ΔE1A, partial ΔE1B, and ΔE3 genetic background using the AdEasy adenoviral vector system (Strategene, La Jolla, CA). The SIVmac239 genes were cloned into the E1 region of the rAd5 vector under the control of the hCMV immediate-early promoter-enhancer and the SV40 stop-polyadenylation signal. All vectors were rescued in HEK 293 cells and purified by double cesium chloride centrifugation. Dosing was based on viral particles (VP), determined by spectrophotometry [45]. We injected 10^11^ VP of each vector intramuscularly (using 27-gauge needles) at three different sites; right shoulder, left forearm and right thigh. Of note, we rotated these sites based on the previous vaccination scheme used for rYF17D so that each site did not receive vectors encoding fragments of the same protein in both vaccinations. We reasoned that this strategy would maximize the dispersal of antigens among lymph nodes and thereby avoid immunodominance.

### IFN-γ ELISPOT Assays

We isolated peripheral blood mononuclear cells (PBMC) from EDTA-treated blood by Ficoll-Paque PLUS (GE Health Sciences) density centrifugation. We used PBMC or PBMC depleted of CD8^+^ cells using magnetic bead separation (Miltenyi Biotec, Auburn, CA), directly in precoated ELISpot^PLUS^ kits (MABTECH Inc., Mariemont, OH) for the detection of monkey IFN-γ according to the manufacturer’s protocols. Briefly, 10^5^ PBMC were used per well and incubated for 14 to 18 hours at 37°C in 5% CO_2_. As a positive control, 5 µg/ml of concanavalin A (Sigma Chemical, St. Louis, MO) was added to the cells, and a set of negative control wells of media only was also included on each plate. Peptide pools of ten 15mers overlapping by 11 amino acids spanning all SIVmac239 proteins included in the vaccine regimens were used at a concentration of 1.0 µM, while minimal optimal peptides corresponding to SIVmac239 or YF17D epitopes were used at a concentration of 10.0 µM. All SIV peptide pools were obtained from the AIDS Research and Reference Reagent Program, Division of AIDS, NIAID, NIH.

Test wells were run with two replicates, while control wells were run with replicates of 2, 4, or 6, depending on the assay. Assay results are shown as spot-forming cells (SFC) per 10^6^ PBMC. Responses comprising <50 SFC per 10^6^ cells were considered negative and were not tested statistically. Positive responses were determined using a one-tailed *t* test and an alpha level of 0.05, where the null hypothesis was that the background level would be greater than or equal to the treatment level. If determined to be positive statistically, the values were reported as the average of the test wells minus the average of all negative-control wells.

### Intracellular Cytokine Staining (ICS) Assay

We performed multi-parameter ICS by incubating freshly isolated PBMC from rhesus macaques with 1.0 µM of separate peptide mixtures spanning the ORFs of Vif, Nef, Tat, and Rev. Vpr and Vpx peptides were combined in one test. We divided the Gag peptides in two tests spanning amino acids 1–291 and 281–510; reactivity to this protein is reported as the sum of these two tests. We used staphylococcal enterotoxin B (SEB) stimulation for our positive control and tissue culture medium devoid of stimulatory peptides for our negative control. We also added anti-CD28 (clone L293; BD Biosciences), anti-CD49d (clone 9g; Pharmigen), and anti-CD107a PE (clone H4A3; BD Biosciences) antibodies and 5.0 µg of Brefeldin A and Golgi Stop (BD Biosciences) to each test. We then placed the samples in a 5.0% CO_2_ incubator at 37°C. After overnight incubation, we stained the cells at room temperature with antibodies directed against the surface molecules CD4 (PerCP Cy5.5; clone L200; BD Biosciences), CD8 (Pacific Blue; clone RPA-T8; BD Biosciences), CD14 (ECD; clone RMO52; Beckman Coulter), and CD19 (ECD; clone JA.119; Beckman Coulter). After 20 minutes, we washed the cells with FACS buffer (10% of fetal bovine serum in PBS 1X) and fixed them with a 2.0% paraformaldehyde solution. After 30 minutes, we washed off the paraformaldehyde and permeabilized the cells with 0.1% Saponin buffer (FACS buffer containing saponin) and stained them with antibodies directed against the intracellular molecules IFN-γ (PE-Cy7; clone 4S.B3; BD Biosciences), TNF-α (Alexa700; clone MAb11; BD Biosciences), IL-2 (APC; clone MQ1-17H12; BD Biosciences), and MIP-1β (FITC; clone 24006; R&D Systems). After a 45-minute incubation, we washed the cells twice with Saponin buffer and fixed them with 2.0% paraformaldehyde. Samples were acquired using FACS DIVA version 6 on a Special Order Research Product (SORP) BD LSR II equipped with a 50 mW 405 nm violet, a 100 mW 488 nm blue, and a 50 mW 640 nm red laser and were analyzed by FlowJo 9.2 (TreeStar, Inc.). Analysis and presentation of distributions were performed using Pestle and SPICE version 5.22023, downloaded from http://exon.niaid.nih.gov/spice [46].

For data analysis, we first gated on forward scatter height (FSC-H) versus forward scatter area (FSC-A) to remove doublets (Fig. S1). Subsequently, we gated on the lymphocyte population and then created a dump channel by excluding CD14^+^CD19^+^ events. At this stage, we separated lymphocyte subsets based on their expression of either CD4 or CD8 (excluding those expressing both markers) and conducted our functional analyses within these two compartments. After making gates for each function (IFN-γ, CD107a, TNF-α, MIP-1β, and IL-2), we used the Boolean gate platform to generate a full array of possible combinations, equating to 32 response patterns when testing five functions (2^5^ = 32). We used three criteria to determine positivity of responses; i) background-subtracted responses had to be at least 2-fold higher than the background itself; ii) Boolean gates for each response pattern had to contain ≥10 events; and iii) response patterns had to be greater than matched values obtained in ICS assays using PBMC from three SIV naïve Indian rhesus macaques under the same stimulation conditions.

### Statistical Analysis

To compare SIV-specific T-cell responses induced by the rYF17D/rAd5 and rAd5 regimens, we performed parametric *t*-tests and nonparametric Wilcoxon-Mann Whitney tests. We performed these tests on raw and log_10_-transformed data. In addition, because some of the cells contained ‘zero values’, a constant equal to 0.5 was added to every count prior to log-transforming the data. The results did not vary significantly across the different statistical tests. The only exception was the comparison of the magnitude of Vif-specific responses between rYF17D/rAd5 and rAd5 vaccinees at week 1 post rAd5. The analysis with the parametric *t*-test yielded a p-value of 0.021, compared to a p-value of 0.06 obtained with the nonparametric Wilcoxon-Mann Whitney test. However, since the data at this time point was highly skewed, we conducted a permutation *t*-test (20,000 samples) on the log-transformed values to serve as an additional sensitivity analysis and obtained a p-value of 0.096. Thus, results from the non-parametric and long-transformed parametric *t*-tests are likely more reliable.

## Results

### Generation of rYF17D Viruses Encoding Fragments of SIVmac239 Gag, Vif, and Nef

**Figure 1:**
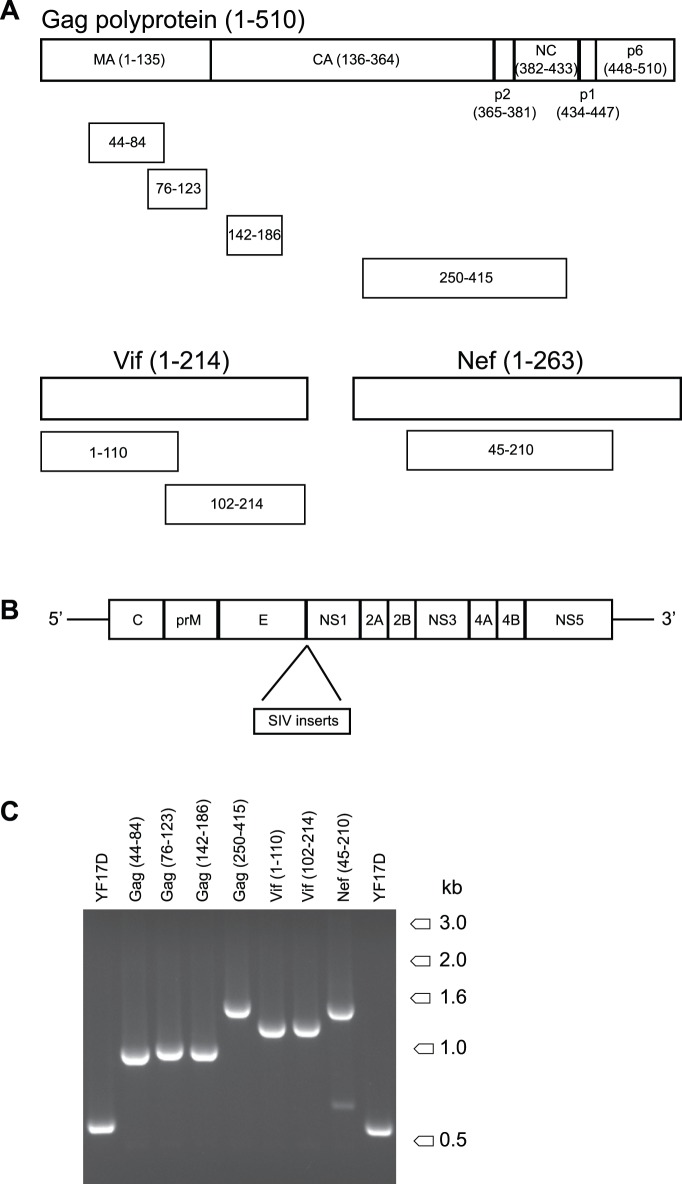
Relative sizes of SIV inserts and genome schematic of rYF17D/SIV vectors. A) Relative size and amino acid position of SIVmac239 minigenes. We designed codon-optimized sequences spanning regions of the Gag, Vif, and Nef proteins. The Gag minigenes covered amino acids 44–84 and 76–123 of Matrix (MA), 142–186 of Capsid (CA), and 250–415 of Capsid, p2, and Nucleocapsid (NC). The Vif ORF was split into two minigenes encoding amino acids 1–110 and 102–214. The Nef sequence spanned the central region of this protein (45–210). B) Genome schematic of recombinant YF17D vaccine vectors. We inserted the SIVmac239 minigenes in the junction between the YF17D E and NS1 genes using a previously described methodology [36]. C) Electrophoretic analysis of RT-PCR amplicons from viral RNA extracted from the seven rYF17D/SIV stocks. The YF17D parental vaccine served as a negative control. We performed these amplifications using YF17D-specific primers flanking the E-NS1 genomic region encompassing the SIVmac239 inserts.

We chose to generate rYF17D vectors expressing fragments of the SIVmac239 Gag, Nef, and Vif proteins – instead of whole ORFs – since large inserts might make rYF17D viruses unstable. Additionally, delivering vaccine antigens as fragments rather than full-length proteins might overcome immunodominance and thereby broaden the repertoire of vaccine-induced T-cell responses [47], [48]. Thus, we split the SIVmac239 Gag precursor protein into four minigenes spanning parts of Matrix (MA), Capsid (CA), p2, and Nucleocapsid (NC); two minigenes encoded the amino and carboxyl termini of the Vif ORF; and one construct covered the central part of Nef (Fig. 1A). We then engineered the backbone of seven different YF17D viruses to express each of these SIV sequences between the E and NS1 genes as described elsewhere (Fig. 1B) [36]. To check the presence of all SIV inserts in their corresponding rYF17D viruses, we extracted vRNA from all rYF17D/SIV stocks and carried out RT-PCR using primers flanking the E/NS1 intergenic region. We obtained amplicons matching the sizes of the SIVmac239 inserts in all rYF17D viruses (Fig. 1C). In the case of rYF17D/Nef(45–210), we noticed a dim PCR fragment of approximately 700 kilobases (Fig. 1C). Since this might be a sign of mixed viral quasi-species, we also sequenced the E/NS1 intergenic region of the rYF17D/Nef(45–210) stock – and the other rYF17D/SIV stocks as well – and confirmed that all SIV inserts were intact (data not shown).

### *In vivo* Replicative Capacity of rYF17D Vectors Encoding SIV Inserts

To determine whether the rYF17D/SIV vectors can replicate *in vivo*, we administered 10^5^ PFU of each virus to seven macaques via the subcutaneous route (Table 1). In parallel, we immunized four animals with all seven rYF17D/SIV viruses given at two different doses; r05028 and rh2138 received 10^4^ PFU while r04137 and r98010 received 10^5^ PFU of each vaccine virus (Table 1). We administered the viruses individually at seven different anatomical sites to maximize their dispersal among lymph nodes and thereby avoid immunodominance [48], [49]. We then collected plasma from all animals at several time points post vaccination (p.v.) and measured viral replication by qRT-PCR. Of note, none of the rYF17D/SIV-vaccinated macaques in this study experienced any adverse events. Despite considerable animal to animal variability in the number of vRNA copies/ml of plasma, all animals that received single rYF17D/SIV vectors had at least one positive viral load, which peaked at around day 7 p.v. (Table 2). In contrast, however, we detected viral replication in only two of the four macaques vaccinated with all of the constructs: rh2138 (852 vRNA copies/ml plasma) at day 7 p.v. and r98010 (175 vRNA copies/ml plasma) at day 3 p.v. Given that only four macaques were immunized with all rYF17D/SIV constructs, it was difficult to assess whether these low levels of viral replication were due to the concomitant administration of multiple rYF17D/SIV viruses or simply to variability among animals. In summary, all seven rYF17D/SIV vaccine viruses replicated in rhesus macaques without causing any observable adverse events.

**Table 2:** Replication of rYF17D/SIV vectors *in vivo* 1.

			Days post vaccination			
Animal ID	Insert (aa position)	Dose (PFU)	3	5	7	14
r04147	Gag (44–84)	10^5^	381	0	216	0
r04170	Gag (76–123)	10^5^	85	131	738	0
r04136	Gag (142–186)	10^5^	0	887	666	0
r05079	Gag (250–415)	10^5^	192	537	2,049	0
r05089	Vif (1–110)	10^5^	0	0	984	0
r04109	Vif (102–214)	10^5^	57	90	119	0
r05070	Nef (45–210)	10^5^	283	433	1,815	0
r05028	All constructs	10^4^ each	0	0	0	0
rh2138	All constructs	10^4^ each	0	0	828	0
r04137	All constructs	10^5^ each	0	0	0	0
r98010	All constructs	10^5^ each	224	0	0	0

1 We measured replication of the rYF17D/SIV viruses using a qRT-PCR assay. Values represent vRNA copies/ml of plasma. The limit of quantitation under standard assay conditions was 45 vRNA copies/ml of plasma.

### Immunogenicity of rYF17D Vectors Encoding SIV Inserts

To measure the immunogenicity of rYF17D/SIV viruses, we obtained PBMC from all vaccinated animals and performed IFN-γ ELISPOT at days 14 and 17 p.v. We used pools of peptides (ten 15mers overlapping by 11 amino acids in each pool) spanning the Gag, Vif, and Nef ORFs of SIVmac239. We adjusted these pools for each macaque depending on the region and SIV protein expressed by the rYF17D viruses. For animals that received all constructs, we included all peptide pools in the IFN-γ ELISPOT assays. To measure CD4^+^ T-cell responses to each of these antigens, we employed the same strategy described above using PBMC depleted of CD8^+^ lymphocytes.

Depending on each animal’s MHC haplotype, we also measured CD8^+^ T-cell responses to the Mamu-A*02-restricted Gag_71–79_GY9, Vif_97–104_WY8, and Nef_159–167_YY9 epitopes using individual minimal optimal peptides [50], [51]. To monitor vector-specific cellular responses, we used four YF17D-derived peptides predicted to bind to Mamu-A*01 according to the MHC pathway algorithm, LTPVTMAEV (LV9_1285–1293_), VSPGNGWMI (VI9_3250–3258_), MSPKGISRM (MM9_2179–2187_), and TTPFGQQRVF (TF10_2853–2862_) [52]. We have recently reported that Mamu-A*01^+^ macaques vaccinated with YF17D and a rYF17D virus expressing a fragment of SIVmac239 Gag recognized these four peptides *in vivo* [37].

**Figure 2:**
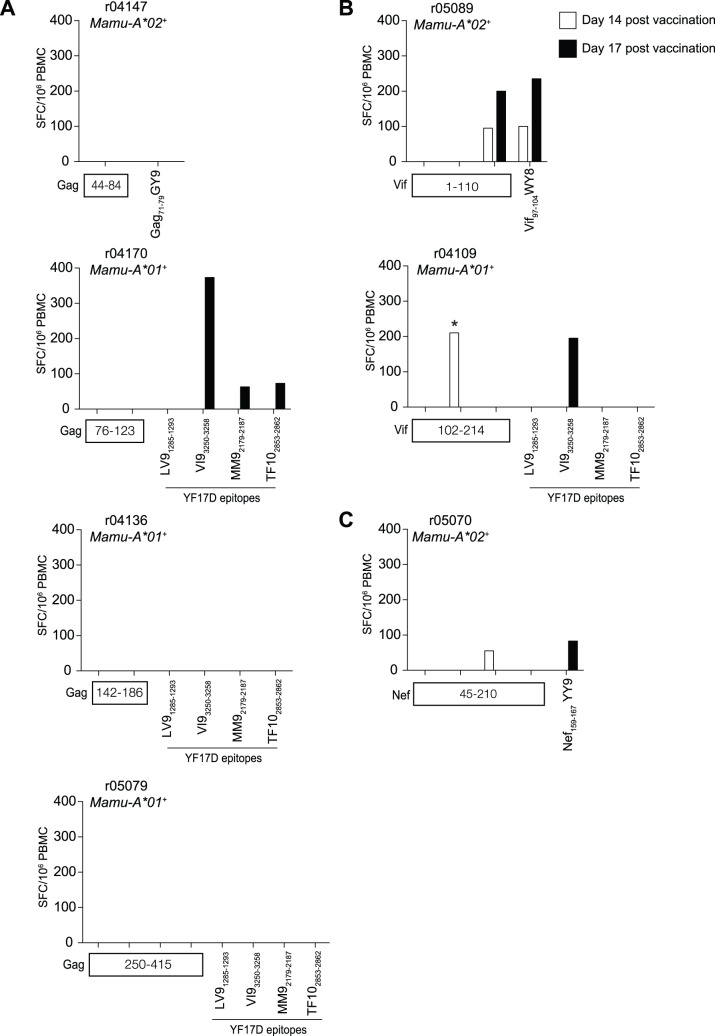
Magnitude of SIV-specific T-cell responses in animals vaccinated with single rYF17D/SIV constructs. We carried out IFN-γ ELISPOT at days 14 (white bars) and 17 (black bars) after the rYF17D vaccination using peptide pools (ten 15mers overlapping by 11 amino acids in each pool) spanning the regions of SIVmac239 Gag, Vif, and Nef encoded in each rYF17D vector. We adjusted these pools for each macaque depending on the region and SIV protein expressed by the rYF17D viruses. Bar graphs indicate the magnitude of IFN-γ-producing cells in PBMC (SFC/10^6^ PBMC) in macaques immunized with rYF17D/Gag constructs (A), rYF17D/Vif constructs (B), and the rYF17D/Nef construct (C). Responses measured at days 14 and 17 following vaccination with the rYF17D/SIV vectors are shown by white and black bars, respectively. Of note, the vaccine candidates rYF17D/Gag(44–84), rYF17D/Vif(1–110), and rYF17D/Nef(45–210) encode three Mamu-A*02-restricted, CD8^+^ T-cell epitopes: Gag_71–79_GY9, Vif_97–104_WY8, and Nef_159–167_YY9, respectively [50], [51]. Since the recipients of these vectors were Mamu-A*02^+^ (r04147, r05089, and r05070; Table 1), we used minimal optimal peptides in their IFN-γ ELISPOT assays to determine the frequency of CD8^+^ T-cells recognizing the Gag_71–79_GY9, Vif_97–104_WY8, and Nef_159–167_YY9 epitopes. We also assessed vector-specific cellular responses by using synthetic peptides corresponding to four Mamu-A*01-restricted CD8^+^ T-cell epitopes in the backbone of YF17D identified in a previous study [37]. The amino acid sequence of these peptides and their corresponding position in the YF17D polyprotein are as follows: LTPVTMAEV (LV9_1285–1293_), VSPGNGWMI (VI9_3250–3258_), MSPKGISRM (MM9_2179–2187_), and TTPFGQQRVF (TF10_2853–2862_). We measured responses to these four epitopes in the Mamu-A*01^+^ macaques r04170, r04136, r05079, and r04109. An asterisk (*) on top of a bar indicates a positive response detected in CD8-depleted PBMC, which likely represents SIV-specific CD4^+^ T-cells. The number of IFN-γ-producing cells in positive control wells stimulated with Concanavalin A at days 14 and 17 were 13,321 and 6,194 SFC/10^6^ PBMC, respectively.

**Figure 3:**
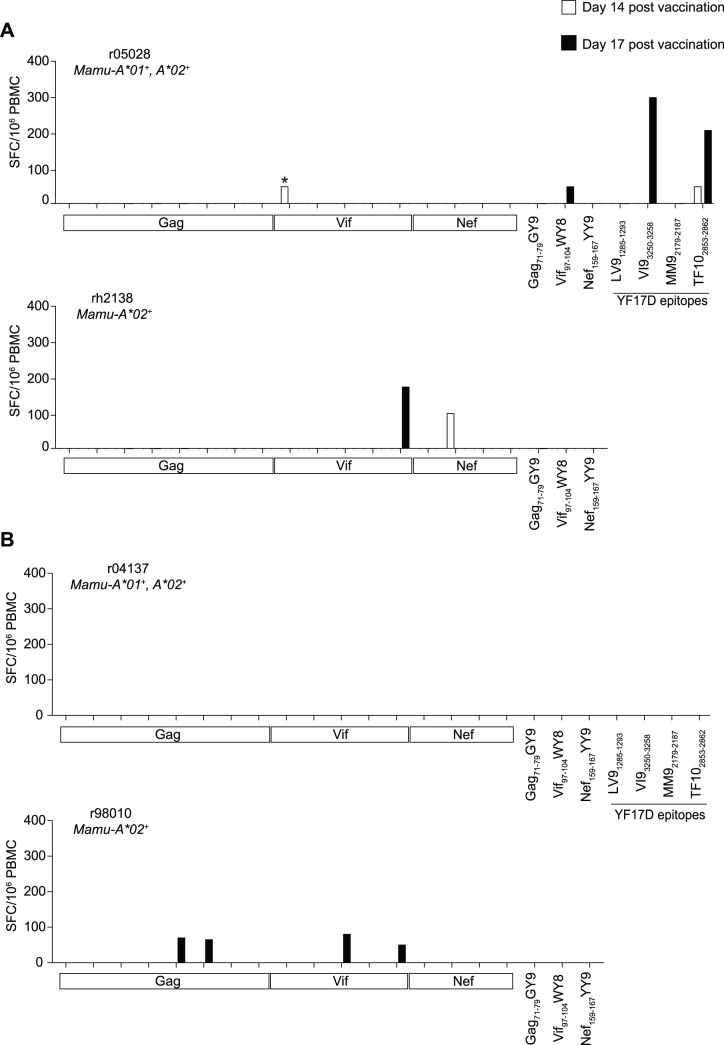
Magnitude of SIV-specific T-cell responses in animals vaccinated with all seven rYF17D/SIV constructs. We used the same approach described in the legend of Figure 2 to measure SIV-specific T-cell responses in animals that were vaccinated with all seven rYF17D/SIV viruses. The only difference was that the IFN-γ ELISPOT assays for these animals contained all peptide pools spanning the regions encoded in the Gag, Vif, and Nef inserts. We performed these analyses at days 14 (white bars) and 17 (black bars) after vaccination with rYF17D/SIV. Vaccinees r05028 and rh2138 received 10^4^ PFU of each vaccine vector (A) while r04137 and r98010 received 10^5^ PFU of each construct (B). Similar to the description in the legend of Figure 2, we measured the frequency of SIV-specific CD8^+^ T-cells in Mamu-A*02^+^ macaques by using minimal optimal peptides corresponding to the three Mamu-A*02-restricted epitopes Gag_71–79_GY9, Vif_97–104_WY8, and Nef_159–167_YY9. We also determined the magnitude of vector-specific CD8^+^ T-cells in the Mamu-A*01^+^ macaques r05028 and r04137 by using synthetic peptides corresponding to the YF17D epitopes LV9_1285–1293_, VI9_3250–3258_, MM9_2179–2187_, and TF10_2853–2862_. An asterisk (*) on top of a bar indicates a positive response detected in CD8-depleted PBMC, which likely represents SIV-specific CD4^+^ T-cells. The average number of IFN-γ-producing cells in positive control wells stimulated with Concanavalin A at days 14 and 17 were 10,980 and 9,269 SFC/10^6^ PBMC, respectively.

Overall, the rYF17D viruses were poorly immunogenic following a single vaccination (Figs. 2 and 3). We could not detect Gag-specific T-cell responses in any of the animals that received single constructs encoding fragments of this protein (Fig. 2A). In contrast, macaques immunized with rYF17D/Vif(1–110), rYF17D/Vif(102–214), and rYF17D/Nef(45–210) developed low frequency T-cell responses to their corresponding SIV inserts (Fig. 2B and C). Among recipients of all seven rYF17D/SIV constructs, the frequency of SIV-specific cellular responses was also low and ranged from undetectable to 178 SFC/10^6^ PBMC in r04137 and rh2138, respectively (Fig. 3A and B). We also noticed that rYF17D/SIV replication *in vivo* did not correlate with their immunogenicity, as evidenced by undetectable Gag-specific T-cells in animals that received constructs encoding fragments of this protein despite measurable replication of these attenuated viruses on at least two time points (Table 2 and Fig. 2A).

Our analysis of vector-specific cellular responses among Mamu-A*01^+^ vaccinees revealed that three macaques recognized the YF17D-derived peptides described above: r04170, who was immunized with rYF17D/Gag(76–123), recognized VI9_3250–3258_, MM9_2179–2187_, and TF10_2853–2862_ (Fig. 2A); r04109 was immunized with rYF17D/Vif(102–214) and recognized VI9_3250–3258_ only (Fig. 2B); and r05028– one of the recipients of 10^4^ PFU of each of the seven rYF17D/SIV constructs – recognized VI9_3250–3258_ and TF10_2853–2862_ (Fig. 3A). These YF17D-specific CD8^+^ T-cell responses were within the same range as those directed against SIV proteins.

It is noteworthy that PBMC from several animals produced significant levels of IFN-γ *in vitro* in the absence of peptide stimulation at the times we carried out the IFN-γ ELISPOT assays (group mean = 94 SFC/10^6^ PBMC; range = 0 to 320 SFC/10^6^ PBMC at day 14 p.v.). As a result, we may have missed low-frequency T-cell responses induced by rYF17D/SIV vaccination since this “spontaneous” production of IFN-γ increased the background of our IFN-γ ELISPOT assays. We have recently described this phenomenon in Indian rhesus macaques immunized with YF17D and found that it is mediated, at least in part, by activated CD8^+^γδ^+^ and CD4^+^ T-cells [53].

In sum, a single immunization with attenuated rYF17D viruses encoding fragments of Gag, Nef, and Vif induced low levels of SIV-specific T-cell responses in approximately 50% of vaccinated macaques.

### Priming with rYF17D/SIV Vectors Increased the Frequency of Vif- and Nef-specific Cellular Responses after a rAd5 Boost

**Figure 4:**
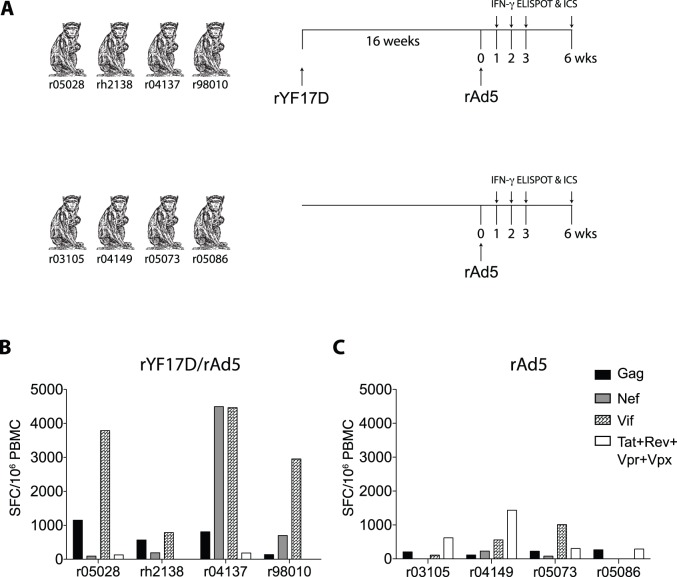
Priming with rYF17D/SIV increased the frequency of SIV-specific T-cell responses after rAd5 boosting. A) Vaccination scheme. Sixteen weeks after vaccination with all seven rYF17D constructs, we boosted r05028, rh2138, r04137, and r98010 with three rAd5 vectors (10^11^ VP each) encoding full-length (i) Gag, (i) Nef, and (iii) Vif fused to Vpr, Vpx, Tat, and Rev. To control for primary SIV-specific T-cell responses induced by these rAd5 vectors, we immunized four SIV naïve macaques (r03105, r04149, r05073, and r05086) with rAd5 alone. We subsequently measured SIV-specific cellular responses in the PBMC of all animals in the weeks following the rAd5 vaccination using IFN-γ ELISPOT and ICS. B and C) Magnitude of SIV-specific T-cell responses (SFC/10^6^ PBMC) to Gag (black bars), Nef (gray bars), Vif (plaid bars), and the combination of Tat, Rev, Vpr, and Vpx (white bars) measured at one week after the rAd5 vaccination of (B) rYF17D/rAd5- and (C) rAd5-immunized macaques. We determined the magnitude of responses to each protein by adding SFC/10^6^ PBMC values obtained in test wells containing peptide pools spanning the SIV inserts described in Figure 1A. The average number of IFN-γ-producing cells in positive control wells stimulated with Concanavalin A at week 1 post rAd5 was 14,259 SFC/10^6^ PBMC.

Previous experiments in mice aimed at testing the immunogenicity of rYF17D in mixed-modality vaccine regimens have shown that rYF17D elicited the highest levels of cellular immune responses when it was given as a prime, but not as a boost (R. Andino, personal communication). We therefore set out to test whether the low-frequency SIV-specific T-cell responses induced by rYF17D vaccination could be boosted by the administration of a heterologous viral vector. To do that, we boosted r05028, rh2138, r04137, and r98010– the four animals that received all seven rYF17D/SIV constructs (Table 1) – with three rAd5 vectors encoding full-length SIVmac239 (i) Gag, (ii) Nef, and (iii) a fusion of the Vif, Vpr, Vpx, Tat, and Rev proteins (Fig. 4A). We performed these vaccinations sixteen weeks after the rYF17D/SIV prime, when the non-specific IFN-γ production in the ELISPOT assay had disappeared and none of the animals had detectable SIV-specific T-cell responses in PBMC (data not shown). To control for primary SIV-specific responses induced by the rAd5 vectors, we immunized four SIV naïve macaques - r03105, r04149, r05073, and r05086– with the same rAd5 constructs described above. We then measured cellular immune responses to all vaccine-encoded SIV antigens in the ensuing weeks using IFN-γ ELISPOT and ICS (Fig. 4A). For sake of simplicity, we reported immune responses directed against Tat, Rev, Vpr, and Vpx as a single group since these antigens were not included in the rYF17D/SIV prime.

We detected robust expansion of SIV-specific cellular responses in three out of four rYF17D/rAd5 vaccinees (r04137, r05028, and r98010) as early as one week after the rAd5 immunization (Fig. 4B). However, there was considerable variability in the antigens targeted by these animals. For instance, r04137 developed high frequency Vif- and Nef-specific T-cells that exceeded 4,400 SFC/10^6^ PBMC whereas r05028 and r98010 focused their T-cell responses mostly on Vif (Fig. 4B). rh2138 targeted Gag, Nef, and Vif similarly but with less abundant responses (Fig. 4B). We also noted that Gag-specific T-cells were a minority among all animals in this group (range: 140 to 1,150 SFC/10^6^ PBMC) (Fig. 4B). By comparison, macaques that received rAd5 alone had detectable responses to Gag, Nef, and Vif at this early time point, but the magnitude of these SIV-specific T-cells did not exceed 1,100 SFC/10^6^ PBMC (Fig. 4C).

**Figure 5:**
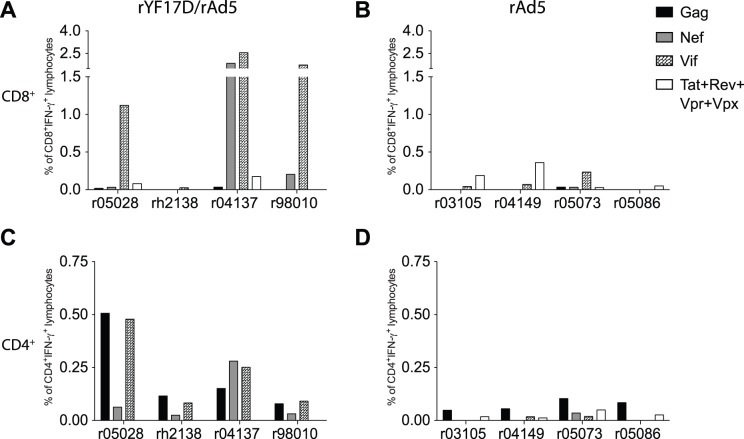
Magnitude of SIV-specific CD4^+^ and CD8^+^ cellular responses in rYF17D/rAd5 and rAd5 vaccinees. At one week after the rAd5 vaccination, we obtained PBMC from all animals and performed ICS to determine the frequency of IFN-γ-producing CD8^+^ (A and B) and CD4^+^ (C and D) lymphocytes specific to Gag (black bars), Nef (gray bars), Vif (plaid bars), and the combination of Tat, Rev, Vpr, and Vpx (white bars). The antigen stimuli in this assay consisted of peptide mixtures spanning (i) amino acids 1–291 of Gag, (ii) amino acids 281–510 of Gag, (iii) the Vif ORF, (iv) the Nef ORF, (v) the Tat ORF, (vi) the Rev ORF, and (vii) the Vpr and Vpx ORFs. Reactivity to the Gag protein is reported as the sum of tests (i) and (ii); reactivity to Tat, Rev, Vpr, and Vpx is reported as the sum of tests (v), (vi), and (vii). A and C show the results for the rYF17D/rAd5 group while B and D show results for the rAd5 group.

Since the IFN-γ ELISPOT data collected after the rAd5 vaccination did not discriminate between CD4^+^ and CD8^+^ cellular responses, we carried out ICS at week 1 after the rAd5 boost to better characterize these vaccine-induced T-cell responses. We found that the high frequency Vif- and Nef-specific T-cells observed in r04137, r05028, and r98010 consisted mainly of CD8^+^ cellular responses (Fig. 5A). In contrast, rAd5-immunized macaques mounted low frequency CD8^+^ cellular responses to all SIV antigens at this early time point (Fig. 5B). Furthermore, rYF17D/rAd5 vaccinees developed considerable levels of CD4^+^ responses, which focused mainly on Gag and Vif and were greater than the CD4^+^ responses measured in animals vaccinated only with rAd5 (Fig. 5C and D).

**Figure 6:**
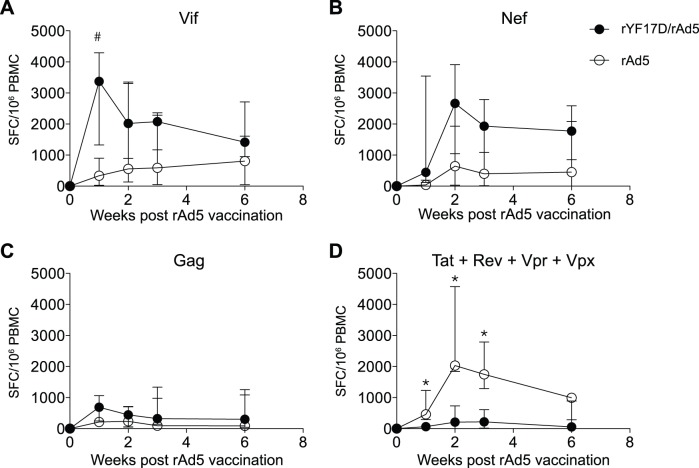
Kinetics of vaccine-induced, SIV-specific T-cell responses in rYF17D/rAd5 and rAd5 vaccinees. We used IFN-γ ELISPOT to determine the magnitude of vaccine-induced T-cell responses at weeks 1, 2, 3, and 6 following the rAd5 immunization. The median frequencies of T-cell responses to Vif (A), Nef (B), Gag (C), and the combination of Tat, Rev, Vpr, and Vpx (D) are shown for rYF17D/rAd5 (black circles) and rAd5 (white circles) vaccinees. We determined the magnitude of responses to each protein by adding SFC/10^6^ PBMC values obtained in test wells containing peptide pools spanning the SIV inserts described in Figure 1A. Error bars represent the interquartile range. Statistical comparisons were made using the Mann-Whitney test. The # symbol in the Vif graph indicates a p-value of 0.06 while asterisks (*) denote p<0.05. The average number of IFN-γ-producing cells in positive control wells stimulated with Concanavalin A at these time points ranged between 4,394 and 14,701 SFC/10^6^ PBMC.

Next, we monitored the kinetics of vaccine-induced T-cell responses to each SIV antigen in the weeks following the rAd5 immunization. In line with the pattern described above, the rYF17D prime increased the magnitude of Vif-specific T-cells in rYF17D/rAd5-immunized animals compared to rAd5 vaccinees, but these differences were not statistically significant (Fig. 6A). The only exception was the peak expansion of Vif-specific T-cells in the rYF17D/rAd5 group at week 1 post rAd5 vaccination, which yielded a borderline difference compared to recipients of the rAd5 regimen (Fig. 6A; p = 0.06). Nef-specific responses also trended higher in rYF17D/rAd5-immunized animals compared to rAd5 vaccinees, but these differences were not statistically significant (Fig. 6B). Gag-specific responses, on the other hand, were similar between the two groups at all time points assessed (Fig. 6C). Strikingly, rAd5 vaccinees developed high frequency T-cell responses against Tat, Rev, Vpr, and Vpx that were significantly higher than those detected in the rYF17D/rAd5 group (Fig. 6D). A breakdown of these responses revealed that they were mostly focused on Tat, more specifically on the immunodominant Mamu-A*01-restricted Tat_28–35_SL8 CD8^+^ T-cell epitope in the case of r03105, r04149, and r05086 (Table 1, data not shown) [54], [55]. Although rYF17D/rAd5 vaccinees r05028 and r04137 are also Mamu-A*01^+^, their Tat_28–35_SL8-specific CD8^+^ T-cell responses were not as abundant as those detected in the rAd5 group (Table 1, data not shown). The reason(s) underlying the immunodominance of Tat_28–35_SL8-specific CD8^+^ T-cells in rAd5 vaccinees but not in rYF17D/rAd5 vaccinees is not entirely clear, but it might be related to a shift in the immunodominance hierarchy toward Vif and Nef epitopes caused by the rYF17D prime.

Together, these results suggest that the rYF17D prime increased the frequency of SIV-specific CD4^+^ and CD8^+^ T-cell responses after rAd5 boosting. However, given the high animal to animal variability observed in this study, a larger adequately powered trial of rYF17D/SIV vaccine candidates may be required to confirm these findings. The brisk expansion of CD8^+^ T-cells targeting Vif, and to a lower extent Nef, seen early after the rAd5 boost might also indicate that rYF17D vectors encoding fragments of these proteins were more effective than the ones encoding segments of Gag at priming SIV-specific T-cell responses.

### Functional Profile of Vaccine-induced T-cell Responses

**Figure 7:**
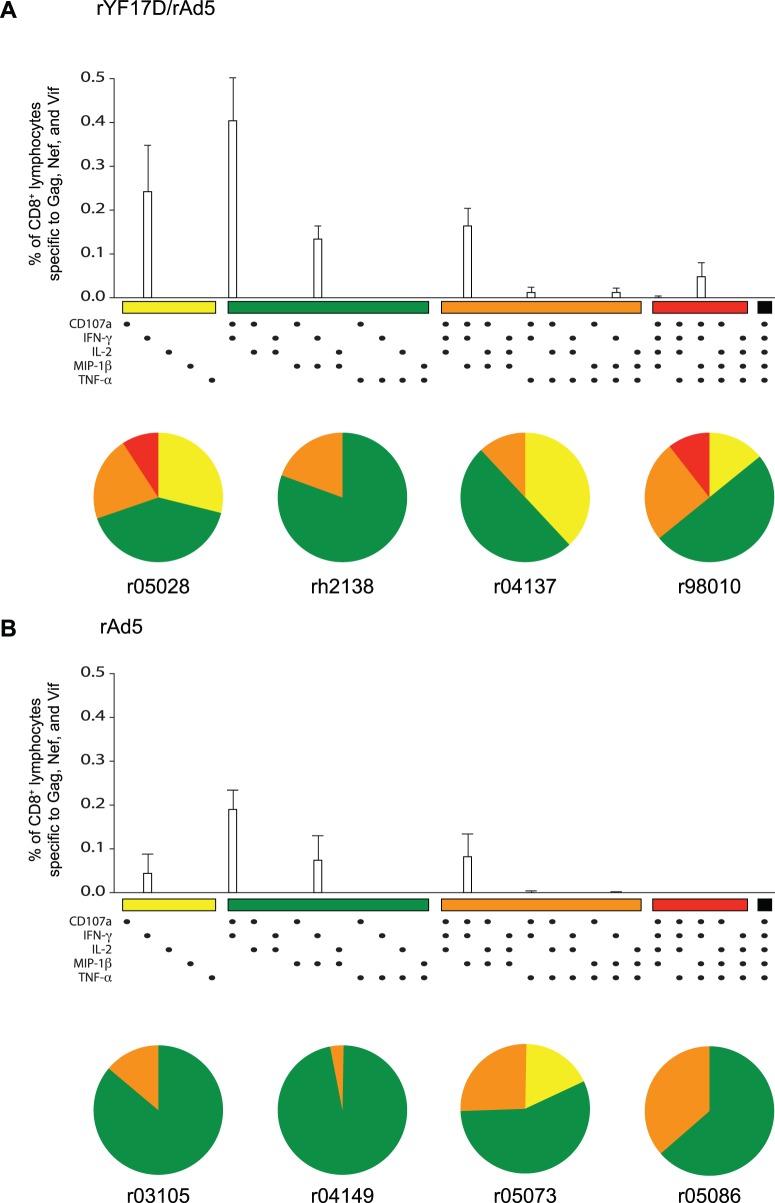
Functional profile of CD8^+^ cellular responses in rYF17D/rAd5 and rAd5 vaccinees. We carried out multi-parameter ICS at week 3 after the rAd5 immunization to determine the ability of SIV-specific CD8^+^ T-cells to degranulate (CD107a) and secrete IFN-γ, TNF-α, MIP-1β, and IL-2. The antigen stimuli in this assay consisted of peptide mixtures spanning (i) amino acids 1–291 of Gag, (ii) amino acids 281–510 of Gag, (iii) the Vif ORF, and (iv) the Nef ORF. Bar graphs indicate the mean total frequency of CD8^+^ lymphocytes specific to Gag, Nef, and Vif capable of producing each combination of functions tested. Pie graphs for each animal indicate the percentage of their CD8^+^ lymphocytes that are specific to Gag, Nef, and Vif and that were positive for one (yellow), two (green), three (orange), four (red), and five (black) immune parameters. A) Functional profile of CD8^+^ cellular responses in rYF17D/rAd5 vaccinees. B) Functional profile of CD8^+^ cellular responses in rAd5 vaccinees. Error bars represent the standard error of the mean.

The ability of HIV-specific CD8^+^ T-cells to degranulate and simultaneously produce multiple cytokines and chemokines has been associated with delayed disease progression in HIV-1-infected individuals [56]. We, therefore, used multi-parameter ICS to determine the functional profile of vaccine-induced CD8^+^ T-cell responses in all animals. Since qualitative features of CD8^+^ T-cells targeting Gag, Nef, and Vif did not vary within each group (data not shown), we decided to compare the total sum of CD8^+^ T-cell responses specific to these three antigens between rYF17D/rAd5 and rAd5 vaccinees at week 3 after the rAd5 vaccination. In agreement with the immunogenicity data presented above, the total frequency of CD8^+^ T-cells recognizing Gag, Nef, and Vif was higher among macaques that were immunized with rYF17D/rAd5 compared to those that received rAd5 only (Fig. 7A and B). We also noticed that the majority of SIV-specific CD8^+^ T-cells in both groups produced IFN-γ either in combination with the degranulation marker CD107a or with MIP-1β (Fig. 7A and B). However, compared to rAd5-immunized animals, rYF17D/rAd5 vaccinees developed SIV-specific CD8^+^ T-cells with a slightly increased functional quality, as seen by higher frequencies of CD8^+^ T-cells staining positive for three and four immune parameters (Fig. 7A and B). We also assessed qualitative features of vaccine-induced CD4^+^ T-cell responses in rYF17D/rAd5 and rAd5 vaccinees. We carried out this analysis at week 1 after the rAd5 boost – the peak expansion of CD4^+^ T-cell responses in both groups (Fig. 5C and D). In addition to secreting IFN-γ, SIV-specific CD4^+^ T-cells in rYF17D/rAd5 vaccinees were also capable of producing IL-2, TNF-α, and MIP-1β in multiple combinations (Fig. S2A). In contrast, rAd5-immunized macaques mounted SIV-specific CD4+ T-cell responses with a more limited functional profile (Fig. S2B). Together, these results suggest that priming with rYF17D improved the functionality of SIV-specific CD8^+^ and CD4^+^ T-cells that expanded after the rAd5 boost.

### Breadth of Vaccine-induced T-cell Responses

**Figure 8:**
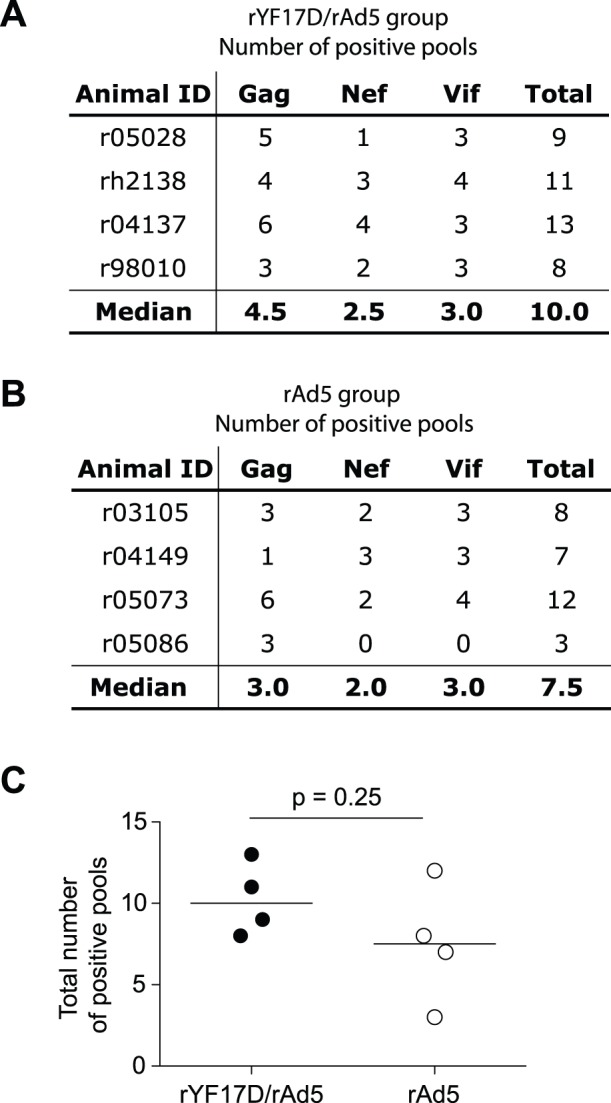
Breadth of vaccine-induced, SIV-specific T-cell responses in rYF17D/rAd5 and rAd5 vaccinees. We determined the breadth of T-cell responses in each animal by adding the number of positive IFN-γ ELISPOT assay responses to peptide pools (15mers overlapping by 11 amino acids) spanning the regions of Gag, Nef, and Vif covered in the SIVmac239 inserts. Panels A and B show the median breadth of T-cell responses to Gag, Nef, Vif, and all three proteins combined in the rYF17D/rAd5 (A) and rAd5 (B) groups. C) Comparison of the total number of peptide pools recognized in rYF17D/rAd5- and rAd5-vaccinated animals. Lines represent the median total number of responses in each group. We used the Mann-Whitney test to determine the p value.

Our final goal was to determine whether priming with rYF17D vectors encoding fragments of SIVmac239 Gag, Nef, and Vif increased the breadth of cellular responses that expanded after the rAd5 boost. We defined breadth as the number of peptide pools spanning the Gag, Nef, and Vif inserts encoded in the rYF17D/SIV vectors for which at least one positive IFN-γ ELISPOT assay response was observed after the rAd5 vaccination. Overall, rYF17D/rAd5-vaccinated animals recognized more peptide pools than did the rAd5 vaccinees (median of total number of pools recognized: 10 versus 7.5, respectively) (Fig. 8A and B). However, the breadth of vaccine-induced T-cell responses in rYF17D/rAd5 vaccinees was not statistically different than that detected in the rAd5 group (p = 0.25; Fig. 8C). Priming with rYF17D/SIV, therefore, did not significantly broaden the repertoire of SIV-specific T-cell responses after a rAd5 boost.

## Discussion

The distinguished safety record of YF17D, coupled to its ability to induce robust and highly functional cellular immune responses in humans make this live-attenuated virus an attractive vector candidate for HIV vaccines. In support of this notion, recombinant live YF17D-based vaccines against other flaviviruses and unrelated pathogens have shown promise in pre-clinical and clinical studies [34], [36], [37], [57]–[66]. We have recently engineered the backbone of YF17D to express amino acids 45–269 of SIVmac239 Gag [37]. This recombinant virus replicated and induced CD8^+^ T-cell responses in Indian rhesus macaques. Franco *et al.* have also demonstrated that a rYF17D encoding HIV Gag p24 induced balanced CD4^+^ and CD8^+^ T-cell responses in BALB/c mice [67]. We, therefore, attempted to expand upon these studies by creating seven new rYF17D viruses expressing fragments of the SIVmac239 Gag, Nef, and Vif proteins. We chose these antigens since vaccine-induced T-cell responses to these proteins have been associated with control of viral replication in a recent SIV efficacy trial [38]. Additionally, a number of studies have linked Gag-specific T-cell responses to lower chronic phase viral loads in HIV-1-infected patients [68]–[70].

We vaccinated a total of eleven Indian rhesus macaques with the rYF17D vectors encoding fragments of SIVmac239 Gag, Nef, and Vif; seven animals received individual constructs while four macaques received all seven rYF17D/SIV vectors. We detected transient viremia in all animals that received single constructs, but only two macaques that were immunized with the seven rYF17D/SIV vectors had positive viral loads. An evaluation of cellular immunity induced by a single vaccination with these vectors revealed low frequency T-cell responses directed against Vif and Nef, while Gag-specific responses were nearly absent. A potential caveat in this analysis relates to the sensitivity of our IFN-γ ELISPOT assay, which might have been reduced since PBMC from these animals produced high background levels of IFN-γ at days 14 and 17 post vaccination. We have recently described this phenomenon, which occurs after YF17D vaccination of rhesus macaques and appears to be caused by the activation of CD8^+^γδ^+^ and CD4^+^ T-cells [53]. Since this “spontaneous” production of IFN-γ increased the background of our ELISPOT assays during the first few weeks after vaccination, we likely missed low-frequency SIV-specific T-cell responses induced by the rYF17D/SIV vectors.

It is also possible that the insertion of SIV sequences in the E/NS1 intergenic region attenuated the rYF17D/SIV constructs even further and thus decreased their immunogenicity compared to the parental YF17D vaccine. Along these lines, a rYF17D virus expressing enhanced green fluorescent protein in this same genomic region demonstrated delayed replication kinetics *in vitro* and induced significantly lower titers of neutralizing antibodies in mice compared to the parental YF17D virus [36]. Furthermore, we have recently evaluated the replication of YF17D and a rYF17D expressing a fragment of SIVmac239 Gag encoding amino acids 45–269 in Indian rhesus macaques using the same qRT-PCR assay employed in this study [37]. We found that positive viral loads came up earlier and at greater magnitudes in animals that received the parental vaccine compared to recipients of the recombinant construct. If lower replication fitness is indeed limiting the magnitude of SIV-specific cellular responses generated by the rYF17D/SIV candidates, additional booster doses might improve the immunogenicity of these vaccine viruses.

It is important to address genetic stability during the development of live-attenuated RNA virus vaccines. In this regard, we have tested the genetic integrity of the rYF17D/SIV viruses by serially passaging them in Vero cells (Bonaldo *et al*., unpublished data). Electrophoretic analysis of RT-PCR amplicons from viral RNA extracted at the 15^th^ passage revealed that four of the seven rYF17D/SIV viruses were stable at this time point. The rYF17D/Nef(45–210) construct yielded a unique gel pattern, containing the amplicon corresponding to the SIV insert and two smaller, less intense fragments. We are currently investigating whether these extra bands are the result of a mixed viral population. We also found that two constructs – rYF17D/Gag(76–123) and rYF17D/Vif(1–110) – lost their inserts at the 10^th^ passage. Although this may explain why we could not detect Gag-specific responses in r04170– the rYF17D/Gag(76–123)-vaccinated macaque, it does not account for the low frequency of SIV-specific T-cell responses induced by the other stable rYF17D constructs. Additionally, rYF17D/Vif(1–110) – one of the genetically unstable viruses – induced detectable Vif-specific cellular responses in r05089 (200 SFC/10^6^ PBMC). Therefore, the rYF17D/SIV vectors were poorly immunogenic even though the majority of these viruses were stable *in vitro*.

Our next step was to test whether the low frequency T-cell responses to Gag, Nef, and Vif induced by vaccination with the rYF17D/SIV constructs could be boosted by a heterologous virus boost. To do that, we immunized eight animals with rAd5 vectors encoding full-length Gag, Nef, and a fusion of the Vif, Tat, Rev, Vpr, and Vpx proteins. Four of the animals had been primed with all seven rYF17D/SIV vectors, while the other four macaques were SIV naïve and served as controls for primary responses induced by the rAd5 (Table 1, Fig. 4A). We found evidence that the rAd5 vaccination boosted SIV-specific cellular responses in animals that had received the mixture of seven rYF17D/SIV vectors, as seen by the robust expansion of Vif-specific T-cells in r05028 and r98010, as well as the high magnitude of Nef-specific T-cells in r04137. On one hand, this is an encouraging finding since it suggests that YF17D – a clinically relevant vector platform for inducing HIV-specific T-cell responses – effectively primed SIV-specific cellular responses and thus is compatible with heterologous prime boost vaccine regimens. On the other hand, the heterogeneity in the magnitude and specificity of the responses that expanded after the rAd5 boost suggests that some rYF17D/SIV constructs were more effective than others at priming SIV-specific T-cells. Furthermore, the immunogenicity of the rYF17D/SIV prime did not predict the expansion of anamnestic responses after the rAd5 boost. Macaque r04137, for instance, had no detectable cellular responses to the SIV antigens following the rYF17D/SIV prime and yet this animal mounted the highest frequency of Vif- and Nef-specific T-cells in the rYF17D/rAd5 group (Figs. 3B and 4B). Conversely, rYF17D/SIV vaccination elicited positive IFN-γ ELISPOT responses to Nef and Vif in rh2138 but this animal did not develop high levels of T-cell responses to these two SIV antigens at week 1 following the rAd5 boost (Figs. 3A and 4B). The reasons for this high animal to animal variability are not entirely clear, but these results suggest that a more thorough investigation of the immunogenicity and *in vivo* replicative capacity of these rYF17D/SIV viruses is warranted, especially when rYF17D/SIV vectors are administered simultaneously.

We also noticed a trend toward broader T-cell responses among rYF17D/rAd5 vaccineees compared to the rAd5 group (median of total number of pools recognized: 10 versus 7.5, respectively). However, this difference did not achieve statistical difference (p = 0.25). The low immunogenicity achieved by the rYF17D/SIV vectors during the priming stage and the small sample size of our experimental groups (n = 4) likely contributed to the comparable T-cell breadth observed in rYF17D/rAd5 and rAd5 vaccinees. Additionally, the fact that rYF17D/rAd5 vaccinees were primed with rYF17D/SIV vectors encoding fragments of Gag, Nef, and Vif and subsequently boosted with rAd5 expressing full-length (i) Gag, (ii) Nef, and (iii) Vif fused to Tat, Rev, Vpr, and Vpx might have restricted the repertoire of vaccine-induced T-cell responses by favoring the expansion of T-cells targeting dominant epitopes, as suggested by previous studies [47]–[49], [71]. Thus, a rYF17D/SIV prime followed by a heterologous virus boost regimen encoding the same SIV minigenes might result in broader SIV-specific T-cell responses.

In summary, the goal of the present study was to evaluate the immunogenicity of live-attenuated rYF17D/SIV viruses expressing fragments of SIV Gag, Nef, and Vif in rhesus macaques. We found evidence that these vaccine viruses replicated *in vivo*, but they engendered low levels of SIV-specific cellular responses. Boosting with rAd5 vectors resulted in robust expansion of SIV-specific T-cells, particularly those targeting Vif and, to a lesser extent, Nef. These anamnestic responses comprised CD4^+^ and CD8^+^ T-cells capable of performing up to four functions after stimulation with synthetic peptides. However, priming with rYF17D/SIV had a limited effect on the breadth of SIV-specific T-cell responses that expanded after the rAd5 boost. It is important to note that these rYF17D/SIV vectors are in their first generation and thus there is still room for improvement. For example, a vaccination regimen comprised of two or three doses of rYF17D/SIV might increase the immunogenicity of these vaccine vectors. In support of this, Santos *et al*. reported that revaccination of YF17D-immune human subjects with the parental YF17D strain resulted in a 3-fold increase in the percentage of activated CD8^+^ T-cells in peripheral blood [72]. Additionally, modification of the SIV inserts increased the genetic integrity of rYF17D/Gag(76–123) and rYF17D/Vif(1–110) – the two recombinant viruses that became unstable after 10 passages *in vitro* (Bonaldo *et al*. unpublished data). We are also testing whether macaques immunized with an improved rYF17D/rAd5 regimen encoding matched SIV minigenes can control viral replication after a pathogenic SIV challenge (Martins *et al*., unpublished data). Optimized rYF17D/HIV vectors may, therefore, be useful for inducing cellular immune responses against the AIDS virus.

## Supporting Information

Figure S1 **Gating strategy for analysis of multi-functional ICS.** A representative example of negative (medium only) and positive (SEB-stimulated) CD8^+^ cellular responses in one rYF17D/rAd5 vaccinee (r05028). These data were obtained at week 3 after the rAd5 boost.
(EPS)

Figure S2 **Functional profile of CD4^+^ cellular responses in rYF17D/rAd5 and rAd5 vaccinees.** We carried out multi-parameter ICS at week 1 after the rAd5 immunization to determine the ability of SIV-specific CD4^+^ T-cells to degranulate (CD107a) and secrete IFN-γ, TNF-α, MIP-1β, and IL-2. The antigen stimuli in this assay consisted of peptide mixtures spanning (i) amino acids 1–291 of Gag, (ii) amino acids 281–510 of Gag, (iii) the Vif ORF, and (iv) the Nef ORF. Bar graphs indicate the mean total frequency of CD4^+^ lymphocytes specific to Gag, Nef, and Vif capable of producing each combination of functions tested. Pie graphs for each animal indicate the percentage of their CD4^+^ lymphocytes that are specific to Gag, Nef, and Vif and that were positive for one (yellow), two (green), three (orange), four (red), and five (black) immune parameters. A) Functional profile of CD4^+^ cellular responses in rYF17D/rAd5 vaccinees. B) Functional profile of CD4^+^ cellular responses in rAd5 vaccinees. Error bars represent the standard error of the mean.
(EPS)

## References

[1] Pneumocystis pneumonia–Los Angeles. MMWR Morb Mortal Wkly Rep 30: 250–252. 6265753

[2] HIV/AIDS JUNPo (2010) Global report: UNAIDS report on the global AIDS pandemic 2010.

[3] McElrath MJ, Haynes BF (2010) Induction of immunity to human immunodeficiency virus type-1 by vaccination. Immunity 33: 542–554. 21029964 10.1016/j.immuni.2010.09.011 PMC3031162

[4] Allen TM, Altfeld M, Geer SC, Kalife ET, Moore C, et al (2005) Selective escape from CD8+ T-cell responses represents a major driving force of human immunodeficiency virus type 1 (HIV-1) sequence diversity and reveals constraints on HIV-1 evolution. J Virol 79: 13239–13249. 16227247 10.1128/JVI.79.21.13239-13249.2005 PMC1262562

[5] Carrington M, O’Brien SJ (2003) The influence of HLA genotype on AIDS. Annu Rev Med 54: 535–551. 12525683 10.1146/annurev.med.54.101601.152346

[6] Friedrich TC, Valentine LE, Yant LJ, Rakasz EG, Piaskowski SM, et al (2007) Subdominant CD8+ T-cell responses are involved in durable control of AIDS virus replication. J Virol 81: 3465–3476. 17251286 10.1128/JVI.02392-06 PMC1866056

[7] Goulder PJ, Watkins DI (2008) Impact of MHC class I diversity on immune control of immunodeficiency virus replication. Nat Rev Immunol 8: 619–630. 18617886 10.1038/nri2357 PMC2963026

[8] Jin X, Bauer DE, Tuttleton SE, Lewin S, Gettie A, et al (1999) Dramatic rise in plasma viremia after CD8(+) T cell depletion in simian immunodeficiency virus-infected macaques. J Exp Med 189: 991–998. 10075982 10.1084/jem.189.6.991 PMC2193038

[9] Matano T, Shibata R, Siemon C, Connors M, Lane HC, et al (1998) Administration of an anti-CD8 monoclonal antibody interferes with the clearance of chimeric simian/human immunodeficiency virus during primary infections of rhesus macaques. J Virol 72: 164–169. 9420212 10.1128/jvi.72.1.164-169.1998 PMC109361

[10] Pereyra F, Jia X, McLaren PJ, Telenti A, de Bakker PI, et al (2010) The major genetic determinants of HIV-1 control affect HLA class I peptide presentation. Science 330: 1551–1557. 21051598 10.1126/science.1195271 PMC3235490

[11] Boaz MJ, Waters A, Murad S, Easterbrook PJ, Vyakarnam A (2002) Presence of HIV-1 Gag-specific IFN-gamma+IL-2+ and CD28+IL-2+ CD4 T cell responses is associated with nonprogression in HIV-1 infection. J Immunol 169: 6376–6385. 12444145 10.4049/jimmunol.169.11.6376

[12] Giraldo-Vela JP, Rudersdorf R, Chung C, Qi Y, Wallace LT, et al (2008) The major histocompatibility complex class II alleles Mamu-DRB1*1003 and -DRB1*0306 are enriched in a cohort of simian immunodeficiency virus-infected rhesus macaque elite controllers. J Virol 82: 859–870. 17989178 10.1128/JVI.01816-07 PMC2224575

[13] Rosenberg ES, Billingsley JM, Caliendo AM, Boswell SL, Sax PE, et al (1997) Vigorous HIV-1-specific CD4+ T cell responses associated with control of viremia. Science 278: 1447–1450. 9367954 10.1126/science.278.5342.1447

[14] Sacha JB, Chung C, Rakasz EG, Spencer SP, Jonas AK, et al (2007) Gag-specific CD8+ T lymphocytes recognize infected cells before AIDS-virus integration and viral protein expression. J Immunol 178: 2746–2754. 17312117 10.4049/jimmunol.178.5.2746 PMC4520734

[15] Liu MA (2010) Immunologic basis of vaccine vectors. Immunity 33: 504–515. 21029961 10.1016/j.immuni.2010.10.004

[16] Barouch DH (2010) Novel adenovirus vector-based vaccines for HIV-1. Curr Opin HIV AIDS 5: 386–390. 20978378 10.1097/COH.0b013e32833cfe4c PMC2967414

[17] Pantaleo G, Esteban M, Jacobs B, Tartaglia J (2010) Poxvirus vector-based HIV vaccines. Curr Opin HIV AIDS 5: 391–396. 20978379 10.1097/COH.0b013e32833d1e87

[18] Robert-Guroff M (2007) Replicating and non-replicating viral vectors for vaccine development. Curr Opin Biotechnol 18: 546–556. 18063357 10.1016/j.copbio.2007.10.010 PMC2245896

[19] Abbink P, Lemckert AA, Ewald BA, Lynch DM, Denholtz M, et al (2007) Comparative seroprevalence and immunogenicity of six rare serotype recombinant adenovirus vaccine vectors from subgroups B and D. J Virol. 81: 4654–4663. 10.1128/JVI.02696-06 PMC1900173 17329340

[20] Buchbinder SP, Mehrotra DV, Duerr A, Fitzgerald DW, Mogg R, et al (2008) Efficacy assessment of a cell-mediated immunity HIV-1 vaccine (the Step Study): a double-blind, randomised, placebo-controlled, test-of-concept trial. Lancet 372: 1881–1893. 19012954 10.1016/S0140-6736(08)61591-3 PMC2721012

[21] McElrath MJ, De Rosa SC, Moodie Z, Dubey S, Kierstead L, et al (2008) HIV-1 vaccine-induced immunity in the test-of-concept Step Study: a case-cohort analysis. Lancet 372: 1894–1905. 19012957 10.1016/S0140-6736(08)61592-5 PMC2774110

[22] Thorner AR, Vogels R, Kaspers J, Weverling GJ, Holterman L, et al (2006) Age dependence of adenovirus-specific neutralizing antibody titers in individuals from sub-Saharan Africa. J Clin Microbiol 44: 3781–3783. 17021110 10.1128/JCM.01249-06 PMC1594810

[23] Hanke T, Goonetilleke N, McMichael AJ, Dorrell L (2007) Clinical experience with plasmid DNA- and modified vaccinia virus Ankara-vectored human immunodeficiency virus type 1 clade A vaccine focusing on T-cell induction. J Gen Virol 88: 1–12. 17170430 10.1099/vir.0.82493-0

[24] Nitayaphan S, Pitisuttithum P, Karnasuta C, Eamsila C, de Souza M, et al (2004) Safety and immunogenicity of an HIV subtype B and E prime-boost vaccine combination in HIV-negative Thai adults. J Infect Dis 190: 702–706. 15272397 10.1086/422258

[25] Russell ND, Graham BS, Keefer MC, McElrath MJ, Self SG, et al (2007) Phase 2 study of an HIV-1 canarypox vaccine (vCP1452) alone and in combination with rgp120: negative results fail to trigger a phase 3 correlates trial. J Acquir Immune Defic Syndr 44: 203–212. 17106277 10.1097/01.qai.0000248356.48501.ff PMC2362395

[26] Barrett AD, Teuwen DE (2009) Yellow fever vaccine - how does it work and why do rare cases of serious adverse events take place? Curr Opin Immunol 21: 308–313. 19520559 10.1016/j.coi.2009.05.018

[27] Monath TP (2005) Yellow fever vaccine. Expert Rev Vaccines 4: 553–574. 16117712 10.1586/14760584.4.4.553

[28] Pulendran B (2009) Learning immunology from the yellow fever vaccine: innate immunity to systems vaccinology. Nat Rev Immunol 9: 741–747. 19763148 10.1038/nri2629

[29] Querec T, Bennouna S, Alkan S, Laouar Y, Gorden K, et al (2006) Yellow fever vaccine YF-17D activates multiple dendritic cell subsets via TLR2, 7, 8, and 9 to stimulate polyvalent immunity. J Exp Med 203: 413–424. 16461338 10.1084/jem.20051720 PMC2118210

[30] Querec TD, Pulendran B (2007) Understanding the role of innate immunity in the mechanism of action of the live attenuated Yellow Fever Vaccine 17D. Adv Exp Med Biol 590: 43–53. 17191376 10.1007/978-0-387-34814-8_3

[31] Barnett ED (2007) Yellow fever: epidemiology and prevention. Clin Infect Dis 44: 850–856. 17304460 10.1086/511869

[32] Organization WH (2009) WHO Vaccine-preventable diseases: monitoring system.

[33] Arroyo J, Miller C, Catalan J, Myers GA, Ratterree MS, et al (2004) ChimeriVax-West Nile virus live-attenuated vaccine: preclinical evaluation of safety, immunogenicity, and efficacy. J Virol 78: 12497–12507. 15507637 10.1128/JVI.78.22.12497-12507.2004 PMC525070

[34] Bonaldo MC, Garratt RC, Caufour PS, Freire MS, Rodrigues MM, et al (2002) Surface expression of an immunodominant malaria protein B cell epitope by yellow fever virus. J Mol Biol 315: 873–885. 11812154 10.1006/jmbi.2001.5258

[35] McAllister A, Arbetman AE, Mandl S, Pena-Rossi C, Andino R (2000) Recombinant yellow fever viruses are effective therapeutic vaccines for treatment of murine experimental solid tumors and pulmonary metastases. J Virol 74: 9197–9205. 10982366 10.1128/jvi.74.19.9197-9205.2000 PMC102118

[36] Bonaldo MC, Mello SM, Trindade GF, Rangel AA, Duarte AS, et al (2007) Construction and characterization of recombinant flaviviruses bearing insertions between E and NS1 genes. Virol J 4: 115. 17971212 10.1186/1743-422X-4-115 PMC2173888

[37] Bonaldo MC, Martins MA, Rudersdorf R, Mudd PA, Sacha JB, et al (2010) Recombinant yellow fever vaccine virus 17D expressing simian immunodeficiency virus SIVmac239 gag induces SIV-specific CD8+ T-cell responses in rhesus macaques. J Virol 84: 3699–3706. 20089645 10.1128/JVI.02255-09 PMC2838090

[38] Martins MA, Wilson NA, Reed JS, Ahn CD, Klimentidis YC, et al (2010) T-cell correlates of vaccine efficacy after a heterologous simian immunodeficiency virus challenge. J Virol 84: 4352–4365. 20164222 10.1128/JVI.02365-09 PMC2863752

[39] Weatherall D (2006) The use of non-human primates in research: A working group report. Final Report December 2006. FRS FMedSci.

[40] Kaizu M, Borchardt GJ, Glidden CE, Fisk DL, Loffredo JT, et al (2007) Molecular typing of major histocompatibility complex class I alleles in the Indian rhesus macaque which restrict SIV CD8+ T cell epitopes. Immunogenetics 59: 693–703. 17641886 10.1007/s00251-007-0233-7

[41] Loffredo JT, Maxwell J, Qi Y, Glidden CE, Borchardt GJ, et al (2007) Mamu-B*08-positive macaques control simian immunodeficiency virus replication. J Virol 81: 8827–8832. 17537848 10.1128/JVI.00895-07 PMC1951344

[42] Caufour PS, Motta MC, Yamamura AM, Vazquez S, Ferreira II, et al (2001) Construction, characterization and immunogenicity of recombinant yellow fever 17D-dengue type 2 viruses. Virus Res 79: 1–14. 11551641 10.1016/s0168-1702(01)00273-8

[43] Cline AN, Bess JW, Piatak MJ, Lifson JD (2005) Highly sensitive SIV plasma viral load assay: practical considerations, realistic performance expectations, and application to reverse engineering of vaccines for AIDS. J Med Primatol 34: 303–312. 16128925 10.1111/j.1600-0684.2005.00128.x

[44] Winstone N, Wilson AJ, Morrow G, Boggiano C, Chiuchiolo MJ, et al (2011) Enhanced control of pathogenic Simian immunodeficiency virus SIVmac239 replication in macaques immunized with an interleukin-12 plasmid and a DNA prime-viral vector boost vaccine regimen. J Virol 85: 9578–9587. 21734035 10.1128/JVI.05060-11 PMC3165762

[45] Mittereder N, March KL, Trapnell BC (1996) Evaluation of the concentration and bioactivity of adenovirus vectors for gene therapy. J Virol 70: 7498–7509. 8892868 10.1128/jvi.70.11.7498-7509.1996 PMC190817

[46] Roederer M, Nozzi JL, Nason MC (2011) SPICE: exploration and analysis of post-cytometric complex multivariate datasets. Cytometry A 79: 167–174. 21265010 10.1002/cyto.a.21015 PMC3072288

[47] Liu Y, Li F, Liu Y, Hong K, Meng X, et al (2011) HIV fragment gag vaccine induces broader T cell response in mice. Vaccine 29: 2582–2589. 21292005 10.1016/j.vaccine.2011.01.049

[48] Rodriguez F, Harkins S, Slifka MK, Whitton JL (2002) Immunodominance in virus-induced CD8(+) T-cell responses is dramatically modified by DNA immunization and is regulated by gamma interferon. J Virol 76: 4251–4259. 11932390 10.1128/JVI.76.9.4251-4259.2002 PMC155093

[49] Im EJ, Hong JP, Roshorm Y, Bridgeman A, Letourneau S, et al (2011) Protective efficacy of serially up-ranked subdominant CD8+ T cell epitopes against virus challenges. PLoS Pathog 7: e1002041. 21625575 10.1371/journal.ppat.1002041 PMC3098219

[50] Loffredo JT, Sidney J, Wojewoda C, Dodds E, Reynolds MR, et al (2004) Identification of seventeen new simian immunodeficiency virus-derived CD8+ T cell epitopes restricted by the high frequency molecule, Mamu-A*02, and potential escape from CTL recognition. J Immunol 173: 5064–5076. 15470050 10.4049/jimmunol.173.8.5064

[51] Vogel TU, Friedrich TC, O’Connor DH, Rehrauer W, Dodds EJ, et al (2002) Escape in one of two cytotoxic T-lymphocyte epitopes bound by a high-frequency major histocompatibility complex class I molecule, Mamu-A*02: a paradigm for virus evolution and persistence? J Virol 76: 11623–11636. 12388723 10.1128/JVI.76.22.11623-11636.2002 PMC136802

[52] Peters B, Bui HH, Sidney J, Weng Z, Loffredo JT, et al (2005) A computational resource for the prediction of peptide binding to Indian rhesus macaque MHC class I molecules. Vaccine 23: 5212–5224. 16137805 10.1016/j.vaccine.2005.07.086

[53] Neves PC, Rudersdorf RA, Galler R, Bonaldo MC, de Santana MG, et al (2010) CD8+ gamma-delta TCR+ and CD4+ T cells produce IFN-gamma at 5–7 days after yellow fever vaccination in Indian rhesus macaques, before the induction of classical antigen-specific T cell responses. Vaccine 28: 8183–8188. 20939995 10.1016/j.vaccine.2010.09.090 PMC3179417

[54] Allen TM, O’Connor DH, Jing P, Dzuris JL, Mothe BR, et al (2000) Tat-specific cytotoxic T lymphocytes select for SIV escape variants during resolution of primary viraemia. Nature 407: 386–390. 11014195 10.1038/35030124

[55] Mothe BR, Horton H, Carter DK, Allen TM, Liebl ME, et al (2002) Dominance of CD8 responses specific for epitopes bound by a single major histocompatibility complex class I molecule during the acute phase of viral infection. J Virol 76: 875–884. 11752176 10.1128/JVI.76.2.875-884.2002 PMC136839

[56] Betts MR, Nason MC, West SM, De Rosa SC, Migueles SA, et al (2006) HIV nonprogressors preferentially maintain highly functional HIV-specific CD8+ T cells. Blood 107: 4781–4789. 16467198 10.1182/blood-2005-12-4818 PMC1895811

[57] Appaiahgari MB, Vrati S (2010) IMOJEV((R)): a Yellow fever virus-based novel Japanese encephalitis vaccine. Expert Rev Vaccines 9: 1371–1384. 21105774 10.1586/erv.10.139

[58] Bonaldo MC, Garratt RC, Freire MS, Galler R (2006) Expression of foreign protein epitopes at the surface of recombinant yellow fever 17D viruses based on three-dimensional modeling of its envelope protein. Cell Biochem Biophys 44: 313–324. 10.1385/CBB:44:3:313 16679518

[59] Guirakhoo F, Pugachev K, Zhang Z, Myers G, Levenbook I, et al (2004) Safety and efficacy of chimeric yellow Fever-dengue virus tetravalent vaccine formulations in nonhuman primates. J Virol 78: 4761–4775. 15078958 10.1128/JVI.78.9.4761-4775.2004 PMC387722

[60] Guy B, Nougarede N, Begue S, Sanchez V, Souag N, et al (2008) Cell-mediated immunity induced by chimeric tetravalent dengue vaccine in naive or flavivirus-primed subjects. Vaccine 26: 5712–5721. 18762226 10.1016/j.vaccine.2008.08.019

[61] Guy B, Guirakhoo F, Barban V, Higgs S, Monath TP, et al (2010) Preclinical and clinical development of YFV 17D-based chimeric vaccines against dengue, West Nile and Japanese encephalitis viruses. Vaccine 28: 632–649. 19808029 10.1016/j.vaccine.2009.09.098

[62] Guy B, Barrere B, Malinowski C, Saville M, Teyssou R, et al (2011) From research to phase III: preclinical, industrial and clinical development of the Sanofi Pasteur tetravalent dengue vaccine. Vaccine 29: 7229–7241. 21745521 10.1016/j.vaccine.2011.06.094

[63] Monath TP, Guirakhoo F, Nichols R, Yoksan S, Schrader R, et al (2003) Chimeric live, attenuated vaccine against Japanese encephalitis (ChimeriVax-JE): phase 2 clinical trials for safety and immunogenicity, effect of vaccine dose and schedule, and memory response to challenge with inactivated Japanese encephalitis antigen. J Infect Dis 188: 1213–1230. 14551893 10.1086/378356

[64] Monath TP, Liu J, Kanesa-Thasan N, Myers GA, Nichols R, et al (2006) A live, attenuated recombinant West Nile virus vaccine. Proc Natl Acad Sci U S A 103: 6694–6699. 16617103 10.1073/pnas.0601932103 PMC1436023

[65] Smith HL, Monath TP, Pazoles P, Rothman AL, Casey DM, et al (2011) Development of antigen-specific memory CD8+ T cells following live-attenuated chimeric West Nile virus vaccination. J Infect Dis 203: 513–522. 21216868 10.1093/infdis/jiq074 PMC3071232

[66] Tao D, Barba-Spaeth G, Rai U, Nussenzweig V, Rice CM, et al (2005) Yellow fever 17D as a vaccine vector for microbial CTL epitopes: protection in a rodent malaria model. J Exp Med 201: 201–209. 15657290 10.1084/jem.20041526 PMC2212788

[67] Franco D, Li W, Qing F, Stoyanov CT, Moran T, et al (2010) Evaluation of yellow fever virus 17D strain as a new vector for HIV-1 vaccine development. Vaccine 28: 5676–5685. 20600494 10.1016/j.vaccine.2010.06.052

[68] Edwards BH, Bansal A, Sabbaj S, Bakari J, Mulligan MJ, et al (2002) Magnitude of functional CD8+ T-cell responses to the gag protein of human immunodeficiency virus type 1 correlates inversely with viral load in plasma. J Virol 76: 2298–2305. 11836408 10.1128/jvi.76.5.2298-2305.2002 PMC135950

[69] Kiepiela P, Ngumbela K, Thobakgale C, Ramduth D, Honeyborne I, et al (2007) CD8+ T-cell responses to different HIV proteins have discordant associations with viral load. Nat Med 13: 46–53. 17173051 10.1038/nm1520

[70] Rolland M, Heckerman D, Deng W, Rousseau CM, Coovadia H, et al (2008) Broad and Gag-biased HIV-1 epitope repertoires are associated with lower viral loads. PLoS One 3: e1424. 18183304 10.1371/journal.pone.0001424 PMC2170517

[71] Liu J, Ewald BA, Lynch DM, Nanda A, Sumida SM, et al (2006) Modulation of DNA vaccine-elicited CD8+ T-lymphocyte epitope immunodominance hierarchies. J Virol 80: 11991–11997. 17005652 10.1128/JVI.01348-06 PMC1676306

[72] Santos AP, Bertho AL, Dias DC, Santos JR, Marcovistz R (2005) Lymphocyte subset analyses in healthy adults vaccinated with yellow fever 17DD virus. Mem Inst Oswaldo Cruz 100: 331–337. 16113878 10.1590/s0074-02762005000300021

